# Skin Brightening and Anti‐Aging Effects of Human Umbilical Cord Wharton's Jelly‐Derived Mesenchymal Stem Cell Conditioned Media and Herbal Extract: A Randomized, Controlled Double‐Blinded Trial

**DOI:** 10.1111/jocd.71265

**Published:** 2026-11-08

**Authors:** Yun‐Ji Lee, Man S. Kim, Yoonsung Lee, Bark‐Lynn Lew, Soon‐Hyo Kwon

**Affiliations:** ^1^ Department of Dermatology Kyung Hee University Hospital at Gangdong, Kyung Hee University College of Medicine Seoul Republic of Korea; ^2^ Translational‐Transdisciplinary Research Center, Clinical Research Institute Kyung Hee University Hospital at Gangdong College of Medicine, Kyung Hee University Seoul Republic of Korea; ^3^ Center for Space Biomedical Sciences NEXUS Institute, Kyung Hee University Yongin Republic of Korea

**Keywords:** anti‐aging, herbal extracts, human umbilical cord Wharton's jelly‐derived mesenchymal stem cell conditioned media, skin brightening

## Abstract

**Background:**

Human umbilical cord Wharton's jelly‐derived mesenchymal stem cell‐conditioned media and herbal extracts have antioxidant, anti‐inflammatory, and regenerative properties that may improve pigmentation and skin aging. However, clinical evidence supporting their combined use in topical formulations remains limited.

**Aims:**

To evaluate the brightening and antiaging effects of a cosmetic formulation containing human umbilical cord Wharton's jelly‐derived mesenchymal stem cell‐conditioned media and mixed herbal extracts in healthy adults with facial pigmentation and photoaging.

**Patients/Methods:**

In a randomized, double‐blinded, vehicle‐controlled study, 36 participants were assigned to either the active formulation group (*n* = 19) or vehicle control group (*n* = 17) and applied the allocated products for 8 weeks. Objective skin parameters, including average melanin, erythema, texture, pore index, and transepidermal water loss (TEWL), were assessed at baseline, week four, and week eight using Antera 3D and Tewameter. Two blinded dermatologists independently evaluated clinical improvement, and participants completed satisfaction questionnaires.

**Results:**

A total of 36 participants were included in the final analysis. At week eight, the active formulation group showed a significantly greater reduction in average melanin level than the control group. Erythema levels showed no significant changes in either group. Skin texture improvements (R_a_ and R_q_ values) were more pronounced in the active group at week eight, as was the reduction in pore index. TEWL decreased in both groups, with no significant between‐group difference. Blinded dermatologists favored the active formulation for clinical improvement, while participant satisfaction scores did not differ significantly between groups. No treatment‐related adverse events occurred.

**Conclusions:**

The cosmetic formulation containing human umbilical cord Wharton's jelly‐derived mesenchymal stem cell‐conditioned media and herbal extract significantly improved pigmentation, texture, and pore appearance compared to the vehicle control over the eight‐week treatment period, supporting its potential for the short‐term management of early photoaging and pigmentation‐related concerns.

## 1 Introduction

Human umbilical cord Wharton's jelly‐derived mesenchymal stem cell‐conditioned media (wjMSC‐CM) contains growth factors, cytokines, and extracellular matrix‐related components that participate in tissue repair and regeneration [1, 2, 3]. These bioactive molecules promote fibroblast proliferation, enhance collagen synthesis, and support re‐epithelialization by influencing intercellular communication within the dermal‐epidermal unit [4, 5]. Additionally, stem cell‐derived factors have been shown to attenuate inflammatory responses and improve epidermal barrier recovery, suggesting their relevance in addressing early aging changes and textural irregularities [5, 6]. Previous studies have highlighted the regenerative capacity of stem cell‐derived materials in various dermatological or wound‐healing contexts [4, 5, 6]; however, their combined application with botanical agents in consumer cosmetic products has not been evaluated.

Herbal extracts contain diverse bioactive compounds, including flavonoids, terpenoids, and phenolic acids, which are known to modulate oxidative stress, inflammatory pathways, and melanogenic signaling in the skin [1]. These phytochemicals can inhibit tyrosinase activity, suppress proinflammatory cytokines, and stabilize reactive oxygen species, thereby improving pigmentation abnormalities and photoinduced dermal alterations [1, 2]. Several plant‐derived ingredients traditionally used in East Asian medicine, such as *Orostachys japonicus, Eclipta prostrata, Zizania latifolia, and Artemisia princeps*, have demonstrated antioxidative and antimelanogenic effects, supporting their potential utility in cosmetic formulations designed to improve skin tone and texture [2, 3].

Although herbal extracts and stem cell‐derived components exhibit activities relevant to depigmentation and skin rejuvenation individually, clinical data on their combined effects are limited. A topical formulation that incorporates both antioxidant phytochemicals and regenerative stem cell‐derived factors may provide complementary benefits for improving uneven pigmentation, surface roughness, and other manifestations of photoaging [2, 5]. However, evidence supporting these combined effects in controlled human trials is insufficient.

In the present study, we investigated the clinical efficacy of cosmetic formulations containing wjMSC‐CM and mixed herbal extracts. Several biophysical and clinical skin parameters were evaluated to determine the brightening, antiaging, and textural improvement effects of the formulation after 8 weeks of application.

## 2 Methods

### 2.1 Study Population

This double‐blind, randomized, vehicle‐controlled clinical study enrolled healthy adults aged 20–65 years with mild‐to‐moderate facial photoaging features, including dyschromia or fine wrinkles. Participants with sensitive skin (a history of adverse reactions to cosmetic products or topical medications), atopic dermatitis, allergic dermatitis, eczema, and other active facial dermatoses were excluded. Individuals with a history of keloid formation; those who had undergone laser therapy, chemical peeling, whitening procedures, or dermabrasion within the previous 6 months; and those who were pregnant or lactating or had an uncertain pregnancy status were also excluded. The sample size was calculated based on the expected between‐group difference in melanin index at week eight. With a two‐sided alpha of 0.05% and 80% power, 17 participants per group were required. Allowing for a 20% dropout rate, a total of 40 participants were enrolled.

This study was conducted in accordance with the Declaration of Helsinki and the International Conference on Harmonization and Good Clinical Practice Guidelines and was reviewed and approved by the Institutional Review Board of the participating institution (KHNMC 2024–02–023‐004). Written informed consent was obtained from all participants prior to their enrollment in the study. We obtained patient consent to publish all photographic materials. The study was prospectively registered with the Clinical Research Information Service (CRIS; KCT0010899) on July 3, 2024.

### 2.2 Treatment

The participants were randomly assigned in a 1:1 ratio to receive either the active or vehicle formulation, and each participant applied the allocated products to the entire face throughout the 8‐week study period. A randomization list was generated by an independent staff member using a computer‐based random sequence and was retained in a secure location until unblinding for statistical analysis. Treatment allocation was concealed from both participants and investigators using participant‐specific coded study products.

Before product application, all the participants gently cleansed their faces using a mild cleanser. They were then instructed to apply the assigned formulation twice daily, according to the standardized regimen described in the study protocol. The active formulation consisted of four products (essence, cream, SMTR, and skin booster) containing wjMSC‐CM and mixed herbal extracts, whereas the control formulation contained the same base components without these active ingredients. Detailed compositions of the topical formulations used in the intervention group are provided in Table S1.

In the morning and evening, the participants applied the essence (two pumps) followed by the cream (one pump). At night, they applied SMTR (2–3 pumps) to areas with pigmentation or fine wrinkles, followed by a skin booster (three pumps) and finally, a mixture of skin booster and essence (2–3 pumps) evenly across the entire face. The participants were instructed to avoid using any other whitening or anti‐aging cosmetic products during the study. The use of broad‐spectrum sunscreen and makeup was permitted only after at least 15 min following the application of the study products. Excessive sun exposure was discouraged during the study period.

The test and control formulations were provided in identical containers with identical appearance, texture, and scent, differing only in the presence or absence of active ingredients. All study products were labeled exclusively with a participant‐specific randomization code to maintain double‐blind conditions for both the investigators and participants.

### 2.3 Assessments

In vivo skin parameters were measured at baseline (visit 1), 4 weeks (visit 2), and 8 weeks (visit 3) after treatment. The participants were instructed to cleanse their faces with a mild cleanser and rest in an ambient environment for at least 15 min prior to each measurement. Antera 3D (Miravex, Dublin, Ireland) was used to quantify wrinkles, texture, pores, depressions/elevations, melanin, hemoglobin, and L*a*b (lightness) parameters on both cheeks at each visit. Measurements obtained from the left and right cheeks were averaged for each participant and used for statistical analysis. Measurements were conducted under standardized lighting conditions, and quantitative values were obtained using automated image‐analysis software. Transepidermal water loss (TEWL) was assessed using a Tewameter (Vapometer, Delfin Technologies Ltd., Finland). TEWL was measured three times on each cheek, and the mean values from the left and right cheeks were subsequently averaged for each participant and used for the analysis. The primary endpoint of the study was defined as the between‐group difference in average melanin level at week eight. Secondary endpoints included the between‐group difference in average melanin level at week four; between‐group differences in erythema, skin texture parameters (Ra and Rq), pore index, and TEWL at weeks four and eight; and participant‐reported satisfaction at weeks four and eight.

At each follow‐up visit (weeks four and eight), the investigator evaluated the improvement based on standardized photographs using a 6‐point clinical improvement scale ranging from 0 (no improvement) to 5 (excellent improvement). At weeks four and eight, the participants completed a questionnaire to assess subjective improvement in overall satisfaction, skin tone, wrinkle improvement, elasticity, pore appearance, moisturization, and texture using a 0–10 scale. Adverse events, including blisters, discomfort, pain, swelling, erythema, irritation, peeling, pruritus, rash, burning sensation, stinging, dryness, and dermatitis, were recorded at each visit.

### 2.4 Statistical Analysis

All statistical analyses were performed using SPSS software (version 20.0; IBM Corp., Armonk, NY, USA). For each outcome measure, within‐group changes between the baseline and follow‐up visits (weeks four and eight) were analyzed using a paired *t*‐test or Wilcoxon signed‐rank test. Differences between the active and vehicle‐control formulations were evaluated using chi‐square tests or equivalent parametric or nonparametric tests based on data distribution. A *p* < 0.05 was set to indicate statistical significance. All results are presented as means ± standard deviations.

## 3 Results

A total of 40 participants were enrolled in this study. Three participants were lost to follow‐up before completing the study, leaving 37 participants who completed all visits. One additional participant was excluded from the final analysis because Antera measurements were missing, resulting in a final population of 36 participants (Figure S1). The mean age was 50.5 ± 9.5 years (ranging from 35 to 65 years). Female participants accounted for 97.2% of the study population. Most participants (22/36, 61.1%) had Fitzpatrick skin type III, while the others had Fitzpatrick skin types II (12/36, 33.3%) or IV (2/36, 5.6%) (Table 1).

**TABLE 1:** Baseline characteristics of the participants.

	Treatment group (*n* = 19)	Control group (*n* = 17)	Total (*n* = 36)
Age, years	49.8 ± 9.7 (range, 35–65)	51.4 ± 9.4 (range, 35–65)	50.52 ± 9.50 (range, 35–65)
Sex			
Male	0 (0%)	1 (5.9%)	1 (2.8%)
Female	19 (100%)	16 (94.1%)	35 (97.2%)
Fitzpatrick skin type			
II	6 (31.6%)	6 (35.3%)	12 (33.3%)
III	12 (63.2%)	10 (58.8%)	22 (61.1%)
IV	1 (5.3%)	1 (5.9%)	2 (5.6%)

### 3.1 Melanin and Erythema

In the treatment group, the average melanin level significantly decreased from baseline at both week four and week eight (44.95 ± 5.20 at baseline to 41.84 ± 4.68 at week four, *p < 0.001*; and to 42.00 ± 4.50 at week eight, *p < 0.001*). In contrast, no significant changes were observed in the control group over time (Figure 1a). Intergroup comparisons showed significantly lower melanin levels in the treatment group at weeks four (*p = 0.007*) and eight (mean difference, −3.71; 95% CI, −6.60 to −0.82; *p = 0.013*) (Figure 1b).

**FIGURE 1:**
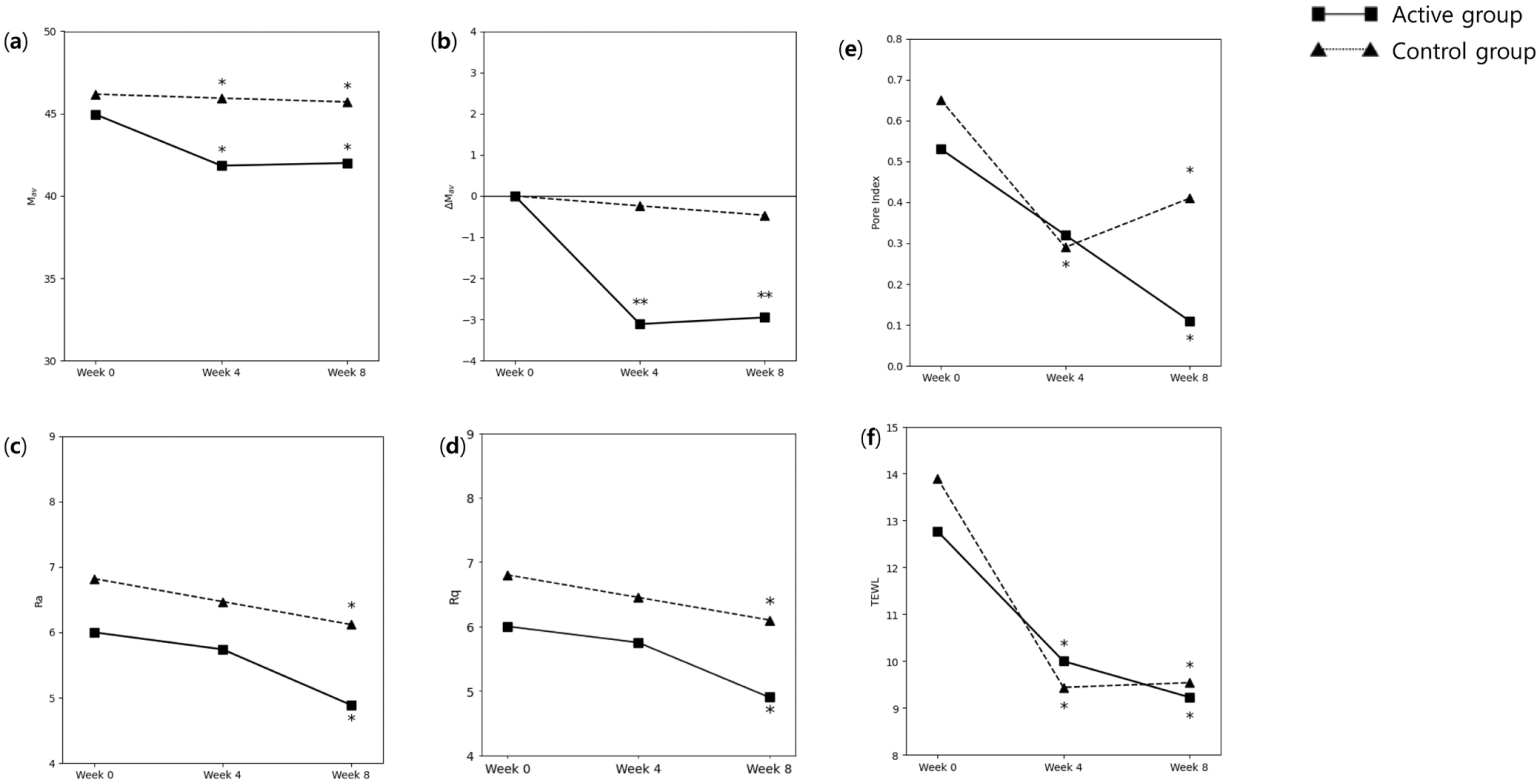
In vivo skin measurements of melanin, texture, pore, and transepidermal water loss: (a) Mav (average melanin); (b) ΔMav; (c) Ra; (d) Rq; (e) Pore; (f) Transepidermal water loss. *p < 0.05 compared to baseline; **p < 0.05 compared to the control group.

The average erythema levels did not change significantly over time in either group and no significant between‐group differences were observed at any time point (*p > 0.05*) (Figure 1c,d).

### 3.2 Texture

Skin texture parameters (*R*_a_ and *R*_q_) significantly improved in the treatment group. In the treatment group, both *R*_a_ and *R*_q_ progressively decreased over time, and the reductions at week eight were statistically significant compared with baseline (*R*_a_: 6.00 ± 1.45 to 4.89 ± 1.29, *p < 0.001*; *R*_q_: 7.74 ± 1.88 to 6.47 ± 1.54, *p < 0.001*) (Figure 2a). Although the control group also showed a modest decrease at week eight, the reduction in *R*_a_ and *R*_q_ was significantly greater in the treatment group than in the control group (*R*_a_, *p = 0.012*; R_q_, *p = 0.031*). These findings suggested that the active formulation improved microtopographical surface irregularities more effectively than the vehicle alone, indicating superior texture refinement.

**FIGURE 2:**
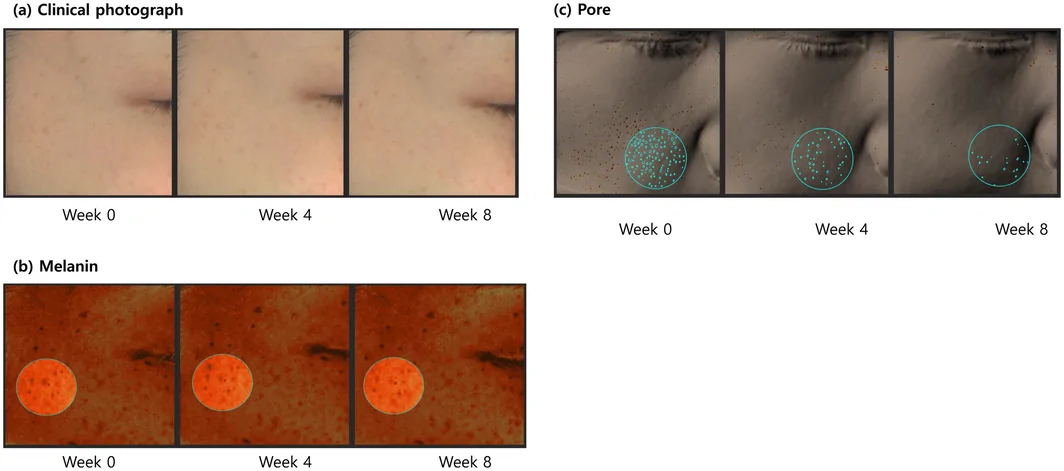
Representative clinical photographs and Antera 3D imaging of skin parameters following 8 weeks of treatment with the active cosmetic formulation. (a) Clinical photographs obtained at baseline, week four, and week eight demonstrating progressive improvement in mottled pigmentation. (b) Representative Antera 3D images of melanin distribution, showing reduction in pigmentation from baseline to week eight. (c) Representative Antera 3D images of pore analysis, indicating decreased pore visibility from baseline to week eight.

### 3.3 Pore

The treatment group showed a significant reduction in pore index at week eight compared with baseline values (0.53 ± 0.51 to 0.11 ± 0.32, *p = 0.007*) (Figure 2b). Meanwhile, the control group demonstrated a significant improvement both at week four (0.65 ± 0.61 to 0.29 ± 0.47, *p = 0.030*) and week eight (0.41 ± 0.51, *p = 0.042*). The between‐group comparison revealed a significantly lower pore index in the treatment group than that in the control group at week eight (*p = 0.035*).

### 3.4 Transepidermal Water Loss

Transepidermal water loss (TEWL) showed a meaningful improvement in the treatment group over the study period. In the treatment group, TEWL significantly decreased at week four (*p = 0.014*) and 8 (*p = 0.004*) compared to baseline (Figure 2c). Similarly, the control group also demonstrated significant reductions in TEWL at weeks four and eight compared with baseline (*p = 0.001* and *p = 0.024*, respectively). Between‐group comparisons revealed no significant differences at any time point.

### 3.5 Investigators' Global Assessment and Patient's Satisfaction Score

The investigators' global assessment demonstrated that a greater proportion of patients achieved good‐to‐excellent improvement in the treatment group than in the control group at weeks four and eight. Overall, the participants were satisfied with the treatment, and the patient‐reported satisfaction scores for overall improvement and individual skin parameters did not differ significantly between the two groups at either time point.

### 3.6 Adverse Events

None of the participants complained of treatment‐related adverse events, such as bleeding, infection, pruritus, paresthesia, allergic reaction, acne, or reactivation of the herpes simplex virus throughout the study period.

## 4 Discussion

In this randomized, double‐blind, vehicle‐controlled clinical study, a topical formulation containing wjMSC‐CM and mixed herbal extracts demonstrated significant improvements in pigmentation and cutaneous surface topography compared to the vehicle control after 8 weeks of treatment. The treatment group exhibited a significantly greater reduction in the average melanin levels at weeks four and eight, whereas erythema showed no significant changes in either group. These findings indicate that the depigmentation effect of the formulation occurred without detectable inflammatory or irritant responses.

Pigmentation is regulated not only by melanocyte activity but also by paracrine signaling within the epidermal‐dermal unit [7]. Stem cell‐derived culture extracts contain multiple bioactive factors, including growth factors, cytokines, and extracellular matrix‐associated proteins, which may influence keratinocyte‐melanocyte interactions and epidermal turnover [4]. Through the modulation of these pathways, wjMSC‐CM may attenuate melanogenesis and facilitate the redistribution of epidermal melanin. Previous studies have suggested that stem cell‐derived culture extracts exert melanogenic effects by downregulating tyrosinase expression and related signaling pathways [8]. Although mechanistic analyses were not performed in this study, the clinical findings were consistent with the proposed biological activities.

In addition to pigmentation improvement, the formulation significantly improved skin surface texture, as demonstrated by reductions in Ra and Rq values. These parameters reflect microtopographical irregularities associated with photoaging‐related dermal structural changes [5, 9]. Stem cell‐derived extracts have been reported to promote fibroblast proliferation, collagen synthesis, and extracellular matrix remodeling [5]. The delayed emergence of intergroup differences at week eight, rather than at week four, is consistent with the slower kinetics of dermal remodeling compared to pigment redistribution [10].

The pore index decreased significantly in the treatment group at week eight. Visible pores are influenced not only by follicular keratinization, but also by perifollicular dermal support and surface roughness [11]. Therefore, the observed pore reduction may represent a secondary effect of improved dermal matrix integrity and surface regularity rather than a direct suppression of sebaceous gland activity. The delayed onset of intergroup differences further supports a remodeling‐dependent mechanism. These findings suggest that improvements in pore appearance may be structurally mediated rather than primarily sebaceous.

The TEWL decreased significantly in both groups without intergroup differences, suggesting that the vehicle formulation itself provided barrier‐supportive moisturizing effects. Therefore, an additional barrier‐enhancing effect specifically attributable to the active ingredients could not be established. Such vehicle‐related improvements are frequently observed in cosmetic trials and may partially obscure incremental barrier recovery attributable to active ingredients [12].

The Investigators' global assessment favored the treatment group, whereas participant‐reported satisfaction did not differ significantly between the groups. Discrepancies between objective instrumental outcomes and subjective perception have been reported in short‐term photoaging studies [13]. Although significant improvements were detected by instrumental and investigator assessments, the absence of a corresponding difference in participant‐reported satisfaction suggests that these changes may not necessarily translate into perceptible clinical benefits from the participants' perspective. Additionally, vehicle‐related improvements in hydration and surface smoothness may have contributed to comparable satisfaction scores between the groups.

No treatment‐related adverse events were observed, confirming the tolerability of the formulation. Together with the stable erythema values, these findings indicate that topical regimens containing wjMSC‐CM may improve pigmentation and skin texture without compromising cutaneous safety.

This study had several limitations. First, the sample size was modest and consisted predominantly of women with Fitzpatrick skin type III and mild photoaging, which limited the generalizability of the results; additionally, potential attrition bias due to incomplete outcome data cannot be excluded. Furthermore, no adjustment for multiple comparisons was applied to the secondary outcomes; therefore, these findings should be interpreted cautiously. The treatment duration was limited to 8 weeks, which may not fully capture the long‐term dermal remodeling effects. However, previous studies commonly evaluated clinical efficacy over a 12‐week period, with substantial and sustained improvements typically observed at the end of the treatment period [14]. In this context, the demonstration of significant improvements within an 8‐week period in the present study is particularly noteworthy. The relatively rapid onset of efficacy may reflect the combined effects of wjMSC‐CM and the herbal extract, although the individual contributions of each component and their potential interactions cannot be determined from the present study. Furthermore, as histological or molecular analyses were not performed, the mechanisms underlying the observed clinical effects remain hypothetical and should be further investigated in future mechanistic studies. Although double‐blinded conditions were maintained using identically presented and coded study products, the success of blinding was not formally assessed. Further studies incorporating larger populations, longer follow‐up study periods, and objective biological markers are warranted to elucidate the underlying mechanisms and durability of the clinical effects.

In conclusion, topical application of a formulation containing wjMSC‐CM and mixed herbal extracts significantly improved pigmentation, skin surface texture, and pore appearance compared to the vehicle control over the 8‐week treatment period, while maintaining a favorable safety profile. These findings support the potential role of bioactive formulations in the short‐term management of early photoaging and pigmentation‐related concerns.

## Author Contributions

Man S. Kim and Yoonsung Lee made substantial contributions to conception and design, and acquisition of data. Bark‐Lynn Lew made significant contributions to conception and study design, and was involved in revising the manuscript critically for important intellectual content. Yun‐Ji Lee and Soon‐Hyo Kwon made substantial contributions to conception and design, acquisition of data, and analysis and interpretation of data. Yun‐Ji Lee, and Soon‐Hyo Kwon are involved in drafting the manuscript, and revising it critically for important intellectual content.

## Funding

The investigational products used in this study were provided by a commercial sponsor, CureNology Co. Ltd.

## Ethics Statement

This study was approved by the Institutional Review Board (IRB) of Kyung Hee University Hospital, Gangdong (IRB No. KHNMC 2024–02–023‐004) on April 4, 2024. Patient consent was obtained at the first visit of the study with signed agreement.

## Conflicts of Interest

The authors declare no conflicts of interest.

## Supporting information


**Figure S1:** Flow diagram of participant enrollment, randomization, follow‐up, and analysis. A total of 40 participants were randomized to either the treatment group (*n* = 20) or the control group (*n* = 20). Three participants were lost to follow‐up, and one participant was excluded due to missing Antera 3D measurements. Finally, 36 participants (19 in the treatment group and 17 in the control group) were included in the final analysis.

**Table S1:** Detailed composition of the topical formulations used in the study.

## Data Availability

The data that support the findings of this study are available from the corresponding author upon reasonable request.
