# Supplementary material for: Skin Brightening and Anti‐Aging Effects of Human Umbilical Cord Wharton's Jelly‐Derived Mesenchymal Stem Cell Conditioned Media and Herbal Extract: A Randomized, Controlled Double‐Blinded Trial
Source: J Cosmet Dermatol. 2026 Nov 8;25(11):e71265. doi: 10.1111/jocd.71265 (PMC13745864; doi:10.1111/jocd.71265)
Supplement: Supplementary file 1 [file JOCD-25-e71265-s001.docx]

Supplementary Table 1. Detailed composition of the topical formulations used in the study

| **Formulation** | **Ingredients, ICID names** |
| --- | --- |
| **Essence** | N&C Mixed Herb Extract^a^; Rosa Damascena Flower Water; Jojoba Seed Oil; Argania Spinosa Kernel Oil; Evening Primrose Oil; Moringa Oleifera Seed Oil; Calophyllum Tacamahaca Seed Oil; Sweet Almond Oil; Avocado Oil; Olive Oil PEG-7 Esters; Hydroxyethylacrylate Copolymer; Sodium Hyaluronate; Panthenol; Opuntia Coccinellifera Fruit Extract; Glycerin; Adenosine; 1,2-Hexanediol; Phenoxyethanol; Rose Flower Oil; Jasminum Officinale Flower Oil; Neroli Oil; Rosewood Oil; Santalum Austrocaledonicum Wood Oil; Lavender Flower Oil; Epidermal Growth Factor; Aloe Barbadensis Leaf Extract |
| **Cream** | N&C Mixed Herb Extract^a^; Jojoba Seed Oil; Argania Spinosa Kernel Oil; Calophyllum Tacamahaca Seed Oil; Evening Primrose Oil; Sweet Almond Oil; Avocado Oil; Moringa Oleifera Seed Oil; Shea Butter; Sorbitan Olivate; Cetyl Alcohol; Olive Oil PEG-7 Esters; Sodium Hyaluronate; Panthenol; Opuntia Coccinellifera Fruit Extract; Glycerin; Adenosine; 1,2-Hexanediol; Phenoxyethanol; Rose Flower Oil; Jasminum Officinale Flower Oil; Neroli Oil; Rosewood Oil; Santalum Austrocaledonicum Wood Oil; Lavender Flower Oil; Epidermal Growth Factor; Rosa Damascena Flower Water |
| **SMTR** | N&C Mixed Herb Extract^a^; Jojoba Seed Oil; Argania Spinosa Kernel Oil; Calophyllum Tacamahaca Seed Oil; Evening Primrose Oil; Sweet Almond Oil; Avocado Oil; Moringa Oleifera Seed Oil; Shea Butter; Beeswax; Allantoin; Tocopherol; 1,2-Hexanediol; Phenoxyethanol; Lavender Oil; Neroli Oil |
| **Skin booster** | N&C Mixed Herb Extract^a^; Human Umbilical Wharton Jelly Mesenchymal Stem Cell Conditioned Media Extract^b^; Jojoba Seed Oil; Argania Spinosa Kernel Oil; Calophyllum Tacamahaca Seed Oil; Evening Primrose Oil; Sweet Almond Oil; Avocado Oil; Moringa Oleifera Seed Oil; Sorbitan Olivate; Cetyl Alcohol; Hydroxyethylacrylate Copolymer; Sodium Hyaluronate; Panthenol; Opuntia Coccinellifera Fruit Extract; Glycerin; Adenosine; 1,2-Hexanediol; Phenoxyethanol; Rose Flower Oil; Jasminum Officinale Flower Oil; Neroli Oil; Rosewood Oil; Santalum Austrocaledonicum Wood Oil; Lavender Flower Oil; Rosa Damascena Flower Water |

^a^ N&C Mixed Herb Extract comprised *Orostachys japonicus* (whole plant), *Eclipta prostrata* (whole plant), *Zizania latifolia* (whole plant), *Portulaca oleracea* (whole plant), *Artemisia princeps* (whole plant), *Eriobotrya japonica* (leaves), *Sasa borealis* (whole plant), and *Dendropanax morbifera* (leaves and stems). The components were mixed at a weight ratio of 0.5–3:0.5–1.5:0.5–1.5:0.5–1.5:0.5–1.5:0.5–1.5:0.5–1.5:0.5–1.5, respectively, and extracted according to the manufacturing method of N&C Medimetic (WO2019132625A1).

^b^Human umbilical cord Wharton’s jelly-derived mesenchymal stem cell-conditioned media (wjMSC-CM), supplied by ROKIT Healthcare, constituted 50% of the Skin Booster formulation. The wjMSC-CM was collected from passage 5 wjMSCs every 3 days for 9 days and sequentially filtered through 0.45-μm and 0.2-μm filters.
