# Supplementary material for: Skin Brightening and Anti‐Aging Effects of Human Umbilical Cord Wharton's Jelly‐Derived Mesenchymal Stem Cell Conditioned Media and Herbal Extract: A Randomized, Controlled Double‐Blinded Trial
Source: J Cosmet Dermatol. 2026 Nov 8;25(11):e71265. doi: 10.1111/jocd.71265 (PMC13745864; doi:10.1111/jocd.71265)
Supplement: Supplementary file 2 [file JOCD-25-e71265-s002.docx]

Supplementary Figure S1. Flow diagram of participant enrollment, randomization, follow-up, and analysis.

A total of 40 participants were randomized to either the treatment group (n = 20) or the control group (n = 20). Three participants were lost to follow-up, and one participant was excluded due to missing Antera 3D measurements. Finally, 36 participants (19 in the treatment group and 17 in the control group) were included in the final analysis.
